# Turkey Hemorrhagic Enteritis (THE): A Short Overview

**DOI:** 10.3390/pathogens13080663

**Published:** 2024-08-06

**Authors:** Laura Musa, Maria Cristina Rapi, Maria Pia Franciosini, Caterina Lupini, Elena Catelli, Maria Filippa Addis, Guido Grilli

**Affiliations:** 1 Department of Veterinary Medicine and Animal Sciences, University of Milan, 26900 Lodi, Italy; laura.musa@unimi.it (L.M.); maria.rapi@unimi.it (M.C.R.); filippa.addis@unimi.it (M.F.A.); 2 Laboratorio di Malattie Infettive degli Animali (MiLab), University of Milan, 26900 Lodi, Italy; 3 Department of Veterinary Medicine, University of Perugia, 06126 Perugia, Italy; maria.franciosini@unipg.it; 4 Department of Veterinary Medical Sciences, University of Bologna, 40064 Ozzano dell’Emilia, Italy; caterina.lupini@unibo.it (C.L.); elena.catelli@unibo.it (E.C.)

**Keywords:** turkey hemorrhagic enteritis virus, immunodepression, secondary infection, THE vaccination, avirulent strains, biosecurity

## Abstract

Turkey Hemorrhagic Enteritis (THE) is an acute disease caused by a *Siadenovirus* that affects 4 week-aged and older turkeys, characterized by acute depression, bloody droppings, and a high mortality rate. The immunosuppressive attributes of THE can protract disease progression and create a predisposition in birds towards subsequent bacterial infectiodoralns involving *Escherichia coli* and *Clostridium perfringens* (necrotic enteritis). Turkey Hemorrhagic Enteritis Virus (THEV) predominantly affects turkeys and carries substantial economic implications for this industry. Macrophages and B lymphocytes are recognized as the predominant target cells for the virus, while the spleen is the principal site of viral replication. Infected cells have also been observed in various other tissues, including the intestines, bursa of Fabricius, cecal tonsils, thymus, liver, kidney, peripheral blood leukocytes, and lungs. The economic relevance of this disease is derived both from the high mortality rate, which can reach 60% depending on the virulence of the strain, and from subclinical disease responsible for poor performance in vaccinated animals. This review aims to provide a comprehensive overview of THE, spanning etiology, epidemiology clinical signs and gross lesions, prevention, and management.

## 1. Introduction

Poultry farming plays a prominent role in the agricultural and food industry, owing to its substantial contribution in terms of income during various stages of breeding, primary and secondary processing, and related industries such as feed production, selection and reproduction, and incubation [1,2]. In the meat production chain, turkeys hold the second position in importance after chickens. The economic outcomes of this industry are strictly dependent on the constant monitoring of prevalent sanitary issues, maintenance of elevated levels of biosecurity, and implementation of prophylactic measures [3].

The genus *Siadenovirus*, within the family *Adenoviridae*, encompasses several species of viruses that pathogenically infect avian species including Turkey Hemorrhagic Enteritis Virus (*Meleagris gallopavo*) (THEV) and Marble Spleen Virus (MSV) of pheasants (*Phasanius colchicus*) [4]. THEV possesses a linear, double-stranded DNA genome consisting of 26.6 kilobase pairs (kb) and encodes eight open reading frames (ORFs) arranged in two clusters [5]. Among these, the hexon and fiber proteins play crucial roles in cell attachment and viral entry, along with their capacity to induce neutralizing antibodies and confer protection against the disease. Siadenoviruses have been reported also in psittacine species: plum-headed parakeet (*Psittacula cyanocephala*), umbrella cockatoo (*Cacatua alba*), budgerigar (*Melopsittacus undulates*), eastern rosella (*Platycercus eximius*), scarlet chested parrot (*Neophema splendida*), cockatiel (*Nymphicus hollandicus*), and red-crowned parakeet (*Cyanoramphus novaezelandiae*) [6,7]. Viral transmission occurs mainly through the oral–fecal route and the main colonization targets are the bursa, intestine, and spleen. Chickens are highly susceptible to THEV infection, which suggests they may act as reservoirs for the virus, potentially influencing its transmission dynamics among turkeys and other avian species [8].

The clinical disease is characterized by depression and the presence of hemorrhagic enteritis. The virus induces the formation of intranuclear inclusions within reticuloendothelial cells. The diagnosis of turkey hemorrhagic enteritis (THE) is based on a range of factors, encompassing clinical symptoms and macroscopic and histological observations, as well as the detection of viral antigens and antibodies through both traditional and molecular methodologies. In cases where clinical and pathological lesions are mild, it is noteworthy to emphasize that the polymerase chain reaction (PCR) technique exhibits superior sensitivity compared to the agar gel immunodiffusion tests for diagnosing THEV infection [9]. The availability of whole genome sequence (WGS) data has facilitated the development of standard, nested, and real-time PCR assays designed for the detection of viral DNA in splenic tissue samples and also from the intestinal content of infected birds [4]. 

To ensure the successful mitigation of THE, it is essential to implement strong biosecurity protocols such as deep physical cleansing, stringent sanitization of feeding equipment, and adequate ventilation [10]. The role of immune cells in the development of clinical signs is unclear [11,12]. However, maternal antibodies are crucial, providing passive immunity to the poults, and protecting them during their first 2–3 weeks of life [13]. 

The economic importance of this disease is due to its potential impact on mortality rates. While the average mortality rate ranges from 5 to 15%, even a 10% mortality rate can cause considerable economic damage in turkey farming. Depending on the pathogenicity of the strain, mortality rates can reach as high as 60% [14]. Furthermore, under field conditions, the immunosuppression following an apparent or asymptomatic HEV infection may manifest as a poor response to vaccinations and/or increased morbidity and mortality from diseases caused by other pathogens. Financial losses attributed to THE in the United States reportedly exceeded USD 3 million annually before the development of a vaccine. Losses related to colibacillosis were estimated at USD 40 million per year. Currently, due to widespread vaccination, highly pathogenic THE outbreaks are rare and generally linked to inadequate vaccination or poor vaccination response [15]. 

**Figure 1:**
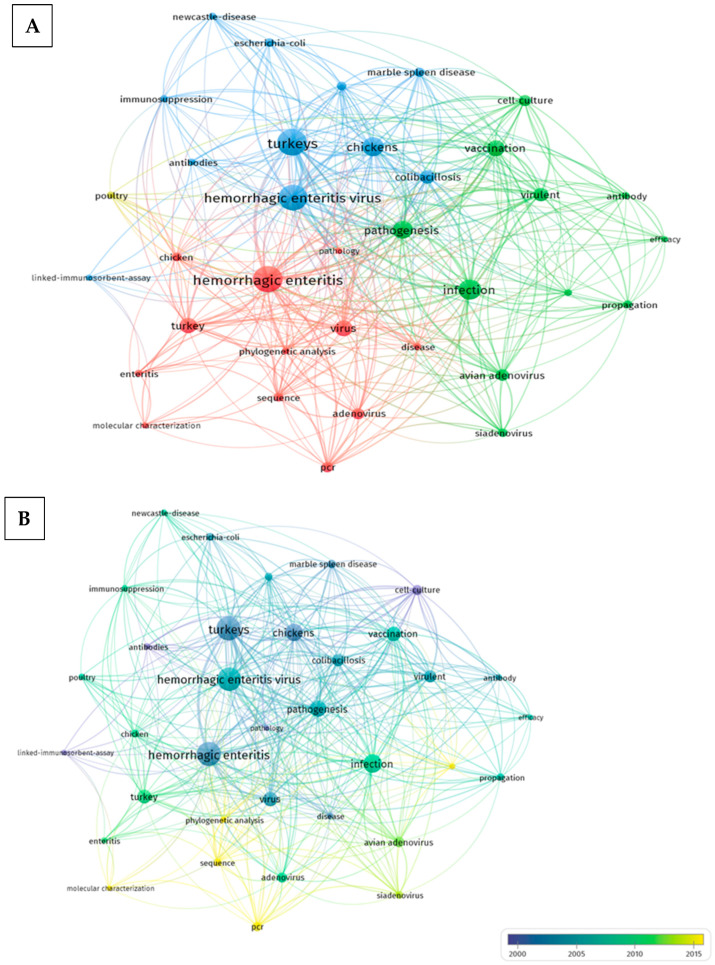
(**A**) Bibliometric network map of scientific research on Turkey Hemorrhagic Enteritis. The network visualization is based on four colored clusters. This figure was created in VOS viewer, University of Leiden, version 1.6.20 (2023) [16], and collected data were obtained from the Web of Science Core Collection and Scopus database using the keywords “Turkey Hemorrhagic Enteritis Virus”. (**B**) Publication map contingent on the respective years of publication, illustrating the variation in publications over time.

A bibliometric network map and a publication intensity map are reported in Figure 1. The map is divided into four colored clusters, each representing a different thematic area or focus within the research (A). The most studied topics are vaccination and immune response; secondary infections and species affected; and classification of the virus and secondary infections or related pathogens. This map shows the distribution of publications related to THEV over time (B). It highlights how the number of publications and the research focus changed across different years. Given the extension of longitudinal studies, this review aims to cover some aspects of THE, highlighting its viral pathogenesis, immune response, and vaccination strategies.

## 2. Historical Background

Turkey Hemorrhagic Enteritis (THE) was initially observed in 1936 and described in 1937 by Pomeroy et al. [17] in Minnesota. By the 1960s, it had spread and was documented as “bankruptcy gut”, affecting turkey farms in Texas and Virginia. The disease was particularly prevalent in both confined and outdoor turkey farms and showed a remarkable tendency to recur in the same locations [18]. The first occurrence of HEV was documented in Europe, specifically in Poland in 1987, originally in 12-week-old turkeys and successively in 16-week-old ones [19]. 

After five consecutive transfers in six-week-old susceptible turkeys, the virus was successfully isolated from both the duodenum and spleen [12]. In 1998, Pitcovski and colleagues successfully sequenced the complete genome of a highly virulent field isolate originating from Israel [5]. The presence of the disease is documented also in chickens, along with splenomegaly and significant reactions of systemic immunity [20,21]. Fasina and Fabricant (1982) reported THEV infection in spleen cell suspension cultures, but could not propagate the virus [22]. In Italy, Marble Spleen Disease (MSD)—caused by a *Siadenovirus* similar to THEV—was first reported by Mandelli et al. [23] in 1977, who also documented THE’s appearance in Italy [23]. Since then, the disease has subsequently spread throughout the country and continues to affect regions where turkeys are industrially raised. In Italy, the virus has been found to cause economic damage to turkey breeders by significantly reducing their egg production [24]. 

According to a study performed in 2013 by the Forlì section of the Istituto Zooprofilattico Sperimentale of Lombardy and Emilia Romagna, in 50 samples collected from turkey farms located in six regions of Northern and Central Italy, seventy-eight percent (78%) were found to be positive for THEV. A mean mortality rate of 13% in males and 8% in females was also reported in the investigated turkey farms [25]. 

## 3. Current Status

Ramsubeik et al. (2023) documented concurrent THEV infections and Necrotic Enteritis (NE) caused by *Clostridium perfringens* type F in four 10-week-old female meat turkeys from a commercial flock in California, with acutely elevated mortality associated with diffuse hemorrhages and petechiae, hyperemia, and necrosis throughout the mucosa of the small intestine [26]. Conversely, the spleen exhibited an increased volume and a speckled appearance. The immunosuppressive impact of THEV, known to be linked to an increase in opportunistic infections, potentially created a vulnerability in the turkeys, leading to an overgrowth of *C. perfringens* and the subsequent development of NE [27,28,29]. Another recent study performed in Australia suggested the presence of subclinical THEV infections, associated with an increase in colibacillosis within commercial turkey populations. However, the prevalence of THE infection in the country remains largely unclear [27]. A study conducted by Gerber (2018) aimed to enhance THEV multiplication in specific-pathogen-free (SPF) chickens. In this study, a total of 562 SPF chickens underwent oral inoculations using an Australian avirulent THEV isolate sourced from turkeys [30]. The study concluded that THEV can be readily propagated in SPF chickens, and conditions to maximize viral retrieval were established.

THEV has been detected in several European countries, including Italy and Hungary. Since the initial recognition of the disease in Hungary in the late 1970s, it has been diagnosed sporadically in its mild form in Hungarian turkey flocks until recently. However, from 2000 to 2005, the number and severity of outbreaks increased significantly, with 9 to 23 affected flocks per year. Most outbreaks occurred in turkeys aged 6 to 8 weeks and were often complicated by *E. coli* infections [9]. In Italy, despite vaccination, THEV continues to circulate. The clinical form of hemorrhagic enteritis is now infrequently observed, but the circulation of immunosuppressive THEV-A strains is suspected [24,31]. This manuscript focuses on countries with the highest turkey meat production and consumption, such as the USA, Canada, and Europe, according to the FAO report. Data on the global prevalence and epidemiology of turkey hemorrhagic enteritis virus remain scarce and are often absent for other countries. This discrepancy likely contributes to the limited reporting and documentation of HEV in regions with lower turkey meat production and trade [32].

## 4. Etiology

The genome of *Adenoviridae* consists of linear double-stranded DNA (dsDNA) ranging from 25 to 48 kb. Recent high-resolution data have shed light on the evolution of adenoviruses, revealing six distinct clusters aligning with recognized genera: *Aviadenovirus*, *Mastadenovirus*, *Atadenovirus*, *Siadenovirus*, *Ichtadenovirus*, and *Testadenovirus* (Table 1) [33]. The classification of species is determined by factors such as phylogeny, genome organization, and restriction fragment length polymorphism. On the other hand, serotyping is carried out through cross-neutralization tests [34]. 

*Siadenovirus* is a genus that includes several species that infect avian species as well as reptiles. It has been hypothesized that siadenoviruses first originated among amphibians and subsequently evolved into avian species [35]. The nomenclature and classification of these viruses as siadenoviruses were based on the noticeable presence of sialidase genes, a distinguishing characteristic that sets them apart from other genera within the family. Fowl adenoviruses (FAdVs) are grouped into the five species Fowl aviadenovirus A to Fowl aviadenovirus E (FAdV-A to FAdV-E), and 12 serotypes (FAdV-1 to FAdV-8a and FAdV-8b to FAdV-11). Among these species, Turkey *Siadenovirus* A (TAdV-A) is specifically identified as type TAdV-3 and is associated with the occurrence of several diseases in various avian hosts, including HE in turkeys [36]. THEV exhibits immunosuppressive properties and is responsible for inducing an acute clinical condition in 4-week or older-aged turkeys, leading to hemorrhagic gastroenteritis and causing relevant economic losses. The lack of vertical transmission is a distinctive characteristic of THEV, setting it apart from other adenoviruses such as egg drop syndrome virus and fowl adenoviruses. 

Current data indicate that recovered birds may be persistently infected and, in some cases, continue to spread the virus for a prolonged period. As an adenovirus, it shows some resistance when protected from desiccation, remaining viable for up to 7 weeks in contaminated carcasses or feces [35,37,38]. This environmental resistance plays a very important role in the persistence of THEV, despite biosecurity practices such as cleaning and disinfection between production cycles. Naturally occurring avirulent strains of THEV (THEV-A) have also been isolated. These strains exhibit a remarkable capacity for efficient replication in turkeys, inducing splenomegaly and immunosuppression, although they do not induce any mortality or lead to the development of intestinal lesions in the host organisms [39,40,41]. In the 1970s, a naturally occurring avirulent strain known as the Virginia avirulent strain (VAS) was discovered among pheasants. VAS has been employed as a live virus vaccine for an extended period and, remarkably, there have been no documented instances of it reverting to a virulent phenotype [39,42]. 

## 5. Pathogenesis 

### THEV Infection and Immune Response

The process of adenovirus entry into a host cell encompasses a sequential series of events and starts with the attachment of the penton base to the integrin receptor, leading to endocytosis and the separation of viral fibers. Membrane lysis is then activated in the early endosome, triggered by low pH, followed by viral interaction with the nuclear pore complex [43]. According to a study conducted by Greber et al. [44], the process of capsid dissociation and DNA release requires the degradation of the structural protein VI by the viral L3/p23 protease [44] that remains inactive in the extracellular virus. Two distinct signals are therefore necessary to initiate this event: the virus must interact with integrin receptors and re-enter an environment with reduced conditions characterized by a lower pH, triggering the activation of membrane lysis, which then allows the viral particles to escape from the endosome into the cytoplasm and proceed to the nucleus [45].

The molecular mechanisms underlying the pathogenesis of viral intestinal diseases in turkeys, including THE, remain unclear [46]. Unlike other adenoviruses such as Fowl adenovirus (*Aviadenovirus*) and egg drop syndrome virus (Duck atadenovirus A), vertical transmission is not observed with THEV. Macrophages and B lymphocytes are considered the primary target cells. Following the process of oral exposure, THEV follows bifurcated pathways in its interaction with the host organism [10]. Primarily, THEV initiates a cycle of replication within B-lymphocytes located in the Bursa of Fabricius. An alternative route involves THEV’s direct migration to the splenic precincts through the peripheral circulatory system. Remarkably, there is a conspicuous influx of CD4+ T-cells and macrophages into the white pulp region, seemingly orchestrated to facilitate viral clearance and/or enhance immune response coordination [47]. This phenomenon underlines the significant presence of splenic hyperplasia that invariably characterizes the acute phase of THEV infection. 

A comparative study in both turkeys and chickens analyzed the immune response against THEV, reporting that the target cells of THEV are believed to be B cells, whereas macrophages are stimulated during infection in chickens but are not as adversely affected or damaged as observed in the case of turkeys. The increased severity of THEV infection in turkeys might stem from prolonged damage to lymphoid tissue and monocyte-like cell populations. This is in part due to the virus replicating extensively in the spleen and the bursa of Fabricius [8,47].

The findings from this study suggest that chickens display a significant susceptibility to THEV infection, leading to the hypothesis that they may potentially serve as reservoirs of infection for turkeys. As anticipated, infected turkeys exhibited specific lesions associated with THEV infection, including spleen enlargement, mottling, and hemorrhagic enteritis. In contrast, infected chickens only displayed splenomegaly. The quantity of THEV-infected cells in the spleen was significantly higher in turkeys compared to chickens, and the macrophages were also stimulated during infection in chickens but were not as damaged as in turkeys [48]. In both species, the immunohistochemical labeling of B-cell surface determinants was reduced, and the splenic B-cell areas became undetectable after THEV infection. Notably, THEV infection triggered elevated nitric oxide production by macrophages in chickens but not in turkeys [19]. Significantly, THEV originating from turkeys exhibited a remarkable ability to propagate in SPF chickens. This investigation led to the identification and implementation of specific conditions to optimize the retrieval of viral content. The most efficacious dose for successful live passage propagation was determined to be seven genome copies (GC), administered to birds aged 9 to 14 days, leading to a noteworthy infection rate of 81%. Additionally, liver and spleen samples from birds infected with THEV at various doses were employed in the development of a prospective vaccine, with a final recovery of 8.6 GC per bird within the inoculation material. These findings underscore significant progress in understanding THEV propagation and advancing vaccine development [30]. Continuing their research, the same research group carried out another study to investigate the feasibility of propagating and quantifying the concentration of THEV within chicken embryos [49]. Overall, no significant variations in post-inoculation mortality rates were observed among the different experimental groups (those sham-inoculated, those inoculated with live THEV, and those inoculated with dead THEV). Regarding the amount of THEV DNA present, the analysis of allantoic fluid collected at 7 days p.i from eggs exposed to live virus indicated a close resemblance to the initial inoculated dose. This finding strongly implies that the propagation of the virus within chicken embryos is not a highly efficient process. This proposition emphasizes the plausible role of chickens in the epidemiological dynamics of THEV transmission among avian species.

As demonstrated in a study conducted by Silim et al. (1981) in turkeys, wherein they tracked the antibody response and the sequential progression of viral antigen in different tissues, THEV antigen was observed in the spleen, liver, intestine, kidney, and bone marrow between 2 to 6 days post-inoculation (p.i.) using immunohistochemical staining (IHC) [42]. The highest viral titers in the spleen were recorded on day 3 p.i. However, no virus was detectable beyond day 6 p.i. The precise mechanism underlying the formation of intestinal lesions during THEV infection remains unclear; however, there is an association with systemic shock induced by T lymphocytes in response to the viral infection, as proposed by Pierson and Fitzgerald [50]. Furthermore, nonstructural proteins such as ORF1 and E3 may potentially play a role in this process. The analysis of numerous strains of THEV has revealed that virulence likely revolves around the complex interplay of multiple factors. Predicting differences in virulence based on genetic markers has proven elusive, as reported by Beach et al. [35], although the same research group pointed out that variations in the sequence of ORF1, E3, and fib in THEV strains with distinct phenotypes strongly suggest that these genes play a critical role in determining virulence. However, modifications in the glycosylation signals within the fiber of most virulent strains suggest their involvement in bolstering virulence. Conversely, alterations detected in the same regions of putative ORF1 and E3 proteins across all avirulent strains might be responsible for functional changes, resulting in decreased virulence. Nonetheless, the in vitro replication of THEV has proven to be quite challenging due to the limited availability of susceptible cells. MDTC-RPl9 cells have emerged as the most suitable host for propagating and isolating both virulent and avirulent THEV [51,52].

## 6. The Impact of THEV on Immune Cells: An Analysis of the Main Effects

B cells and macrophages have consistently emerged as the main cellular targets for THEV infection. Studies conducted in the early stages have shown that chemical bursectomy is an effective method for protecting turkeys from the lesions and mortality caused by THEV infection. Cyclophosphamide-induced B cell depletion has been found to significantly inhibit THEV replication in the spleen of infected turkeys, as reported in several studies [8,53]. Further flow cytometric analysis has revealed a decrease in the proportion of IgM+ B lymphocytes in both the spleen and peripheral blood of THEV-infected turkeys on days 2, 3, and 9 post-infection. The destruction of B cells in response to THEV infection is believed to occur via necrosis and apoptosis, as supported by immunohistochemical studies demonstrating a reduced or an absent expression of B cell surface markers in THEV-infected turkeys, potentially due to an apoptotic process [54]. The specific role of T cells in THEV infection is still unclear. Initial studies revealed that THEV infection causes a temporary suppression in the mitogenic response of spleen cells [17,55], potentially contributing to immunosuppression. Flow cytometric analysis has shown an increase in CD4+ cells in the spleen on days 4–6 post-THEV infection, and elevated CD8+ cells are observed only on day 16 post-infection. The reasons for lymphocyte alterations and their significance are not fully understood, but this rise in CD8+ cells might be associated with viral clearance [17,56].

It has been demonstrated that THEV infects macrophages, leading to a reduction in their functional capabilities as they may undergo necrosis and apoptosis, compromising their antigen-presenting function. As an indication of compromised macrophage function, a study disclosed that, during the peak of infection, splenic macrophages in turkeys infected with THEV failed to generate nitric oxide (NO) following ex vivo stimulation with lipopolysaccharide (LPS) [57]. Extensive research on various avian and mammalian viruses suggests that NO serves as a host-defense mechanism on day 16 post-infection and plays a key role in destroying infected cells, thereby preventing the spread of infectious agents [58]. In THEV-infected birds, the reduced ability to produce optimal levels of NO by macrophages, the primary line of defense against secondary infections, may increase susceptibility to bacterial and other viral infections.

## 7. Clinical Signs

This virus primarily affects turkeys aged between 4 and 11 weeks, with the highest incidence occurring at 7 to 9 weeks of age [48]. Being an adenovirus, it possesses robust environmental resistance and can be introduced into flocks through various vectors, including both living and non-living carriers. Once it infiltrates a flock, THEV spreads rapidly, often with a 100% morbidity rate. It follows an oral–fecal cycle, being shed in feces, which contributes largely to its rapid dissemination. Despite mortality rates being relatively low, ranging from 5% to 15%, it is crucial to recognize that even a 10% loss can result in substantial economic repercussions in turkey farming [11]. Depending on the virulence of the strain, mortality can reach as high as 60%. Interestingly, when a population encounters this virus for the first time, initial mortality rates tend to be very high, but its pathogenicity tends to decrease over time. 

Clinical symptoms are often undetectable and affected animals typically undergo spontaneous death despite being in good nutritional condition. Classical acute forms characterized by a typical symptomatologic pattern are uncommon in industrialized regions, whereas immunosuppressive forms, often associated with scarring and secondary infections, are more prevalent. Hemorrhagic feces are not commonly observed. Surviving animals are immunosuppressed and more susceptible to secondary infections for several weeks [59]. 

The disease follows an acute course due to the severity of the lesions. Deceased birds exhibit pallor, and the feathers around the cloaca are stained with dark feces due to the digestion of blood. Lower levels of glucose, albumin, and total protein in the blood can also be detected [60]. 

## 8. Macroscopic and Microscopic Lesions

**Figure 2:**
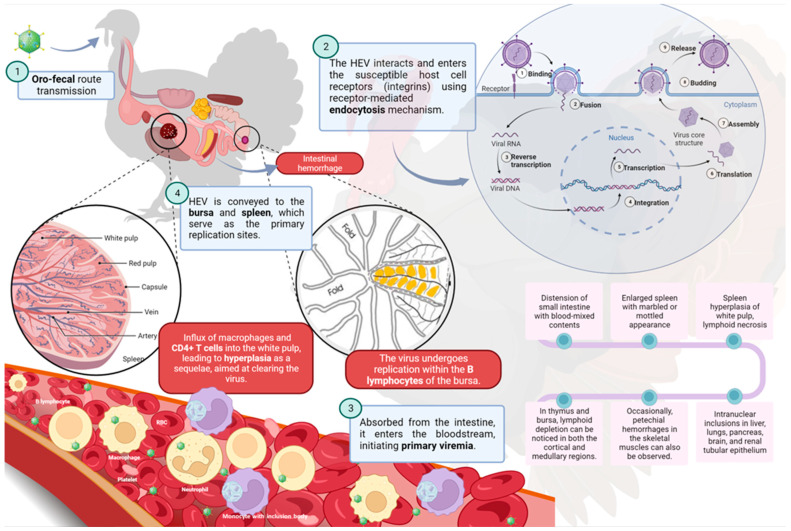
Comprehensive representation of dissemination, pathogenesis, and microscopic /macroscopic findings of Turkey Hemorrhagic Enteritis Virus in infected turkeys. Created with BioRender.com, 2024 BioRender Canada.

This chapter provides a comprehensive examination of both macroscopic and microscopic findings, as illustrated by Figure 2.

### 8.1. Macroscopic Findings

**Figure 3:**
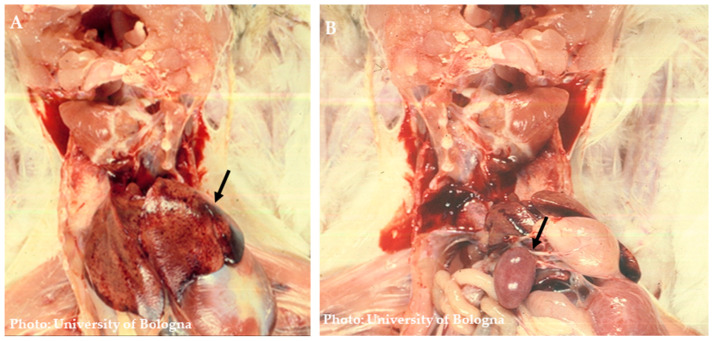
(**A**) Liver with petechial hemorrhages; (**B**) Enlarged, friable, and mottled spleen upon palpation.

**Figure 4:**
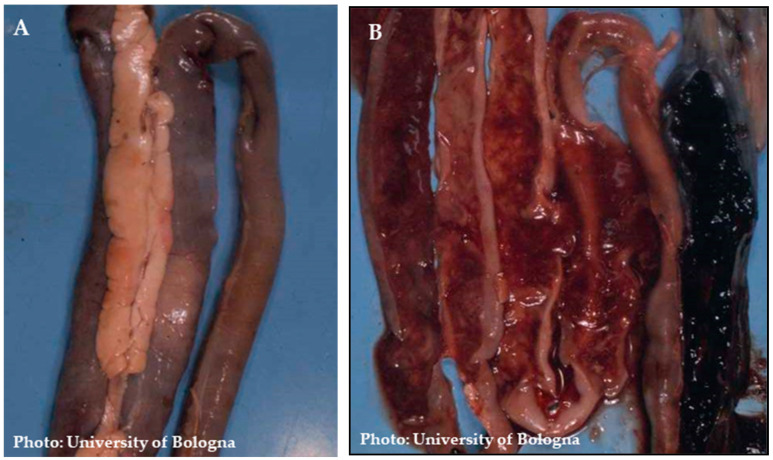
(**A**) Small intestine filled with bloody content (duodenal loop); (**B**) Lesions can be distal in severe cases.

Most of the macroscopic lesions are localized in the spleen and intestine (Figure 2). Among the associated lesions, abnormalities in the spleen are the most consistently observed, and the highest viral load is typically found within the spleen (Figure 3). A significant disparity in the appearance of the spleen can be observed [61,62]; at times it appears pale and smaller in size, while at other times it is visibly enlarged, intensely congested, and can display a purplish hue and grayish necrotic foci (Figure 3). Moderate and severe levels of splenomegaly are commonly linked with hypertrophy of the white pulp [63]. The intestines can be notably distended and filled with unclotted blood (Figure 4) [61]. A study carried out by Itacura et al. [63] documented the successful experimental transmission of the infection to turkeys, comparing the severity of the infection with naturally infected birds and also providing a comprehensive report of the subsequent clinical manifestations and pathological findings observed in infected turkeys. At 3 days post-inoculation (p.i), mild congestion was noted in the small intestines. From 5 to 9 days p.i, distension, slight congestion, and an increase in mucous content were observed in the small intestine. Notably, multiple hemorrhages were seen in the anterior half of the small intestine in only 1 bird out of 23 at 5 days p.i. Additionally, a mild form of chronic enteritis was detected between 10 to 16 days p.i. The gross and histological lesions observed in the experimental cases were consistent with those encountered in natural outbreaks. In severe cases, the intestinal lesions exhibited greater prominence in the duodenal region and extended toward the caecum, causing a severe involvement of this anatomical segment [64]. Death typically occurs due to extensive gastrointestinal (GI) hemorrhages, which can result in the loss of approximately 60% to 70% of the bird’s total blood volume. Occasionally, petechial hemorrhages in the skeletal muscles can also be observed. The incidence of lesions associated with HE is directly correlated with the dosage of the inoculum administered. However, it is important to note that the incubation period remains unaffected by variations in the inoculum dosage. No macroscopic signs indicative of disease are observed in the other organs [59,63].

### 8.2. Microscopic Findings

The characteristic histopathological alterations linked to THE are most prominently exhibited within the immune and gastrointestinal systems [15]. Splenic abnormalities frequently involve hyperplasia of the white pulp and lymphoid necrosis. Furthermore, distinctive basophilic Cowdry type B intranuclear inclusions (INI) can be detected within mononuclear cells, particularly within macrophages and lymphocytes [15]. There is a notable increase in the abundance of large mononuclear cells and plasma cells at various stages of development around the white pulp and sheathed arteries. The most remarkable characteristic is the presence of cells with nuclei exhibiting a ballooned appearance. These nuclei can also display a pale pink to nearly colorless staining, with eccentrically marginated chromatin [61]. The monocytic inflammatory cells within the lamina propria can display nuclei with a ballooned morphology similar to those noticed in the spleen. Virus-positive cells are consistently identified in the lamina propria of the intestine, but not in mucosal epithelial cells. Consequently, it has been proposed that the degeneration and sloughing of intestinal epithelial cells are not attributed to the direct effects of the virus, but rather to an immune system-mediated phenomenon [47]. 

**Figure 5:**
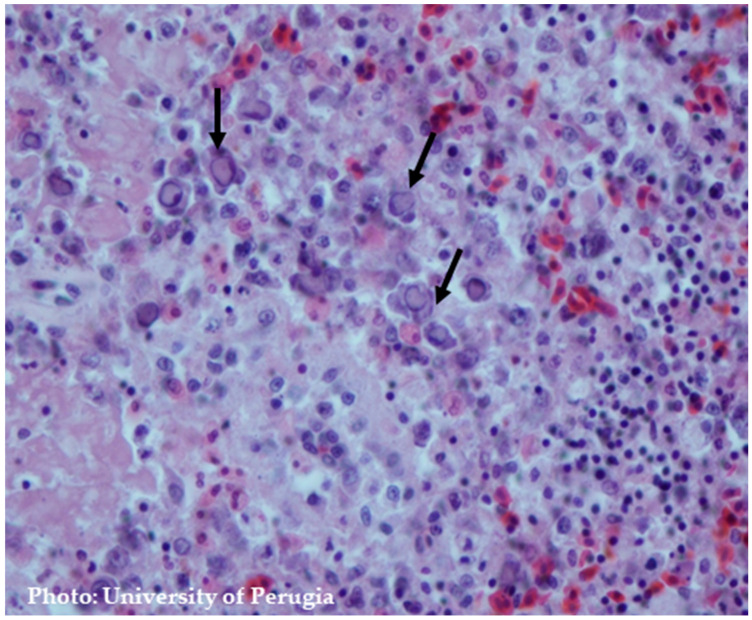
Splenic lesions. Hyperplasia of white pulp and lymphoid necrosis and intranuclear inclusion in lymphoreticular cells with chromatin margination. H&E staining.

The emergence of intestinal lesions following infiltration of the lamina propria of the small intestine with lymphoreticular or lymphoid cells supports the hypothesis that immune cells may play a role in the pathogenesis of HE (Figure 5) [47,65]. Activated T cells may be key in causing intestinal lesions from THEV and leading to hemorrhagic shock in turkeys. The treatment of turkeys with Cyclosporin A (CsA) protects against THEV-induced intestinal hemorrhages by depleting and impairing T cells. In one study, CsA-treated turkeys showed no presence of intestinal hemorrhagic lesions compared to five out of ten in the untreated group. The CsA did not impact the severity of THEV-induced splenomegaly or viral replication [66]. 

## 9. Diagnosis

When contemplating a differential diagnosis for THEV, it is imperative to systematically exclude alternative conditions that may exhibit analogous symptoms and/or impact turkey populations. One potential differential diagnosis could be necrotic enteritis caused by *Clostridium perfringens*. Enlarged and congested spleens in turkeys are also frequently misattributed to THE; however, they commonly arise due to bacteremia associated with organisms such as *E. coli*, *Salmonella* spp., and *Pasteurella multocida*. 

Gastrointestinal hemorrhage and mucosal hyperemia may be linked to acute viral agents, such as highly pathogenic avian influenza virus (HPAIV), Newcastle disease virus, avian reovirus, and parasitic infections induced by various *Eimeria* species (coccidiosis). Splenic enlargement and mottling in the absence of demonstration of MSDV or avian adenovirus splenomegaly (AASV) should prompt histopathologic evaluation for neoplastic diseases such as Marek’s disease, lymphoid leukosis, or reticuloendotheliosis [10,18]. To attain a precise diagnosis of THEV or any other ailment, comprehensive laboratory assessments may be essential, including virus isolation, molecular diagnostic techniques (qPCR), and post-mortem examinations. For THEV isolation, the lymphoblastoid B-cell line derived from turkeys, known as MDTC-RP19, can be employed. Alternatively, if the cell line is unavailable, the virus can be propagated in naive turkeys at 6 weeks of age by inoculating either intestinal contents or splenic material through either the oral or intravenous (IV) route. 

## 10. Vaccination Strategies 

To effectively prevent THE, it is crucial to implement biosafety plans that include thorough physical cleaning of breeding facilities and complete litter removal. Nevertheless, achieving total eradication of THE in industrial poultry production can be a real challenge. In such cases, vaccination remains the only effective option to prevent infection and mitigate subsequent economic losses caused by secondary agents. The optimal time for administering live THEV vaccines is approximately 28 days post-hatch. However, the presence of maternally derived antibodies may interfere with vaccine efficacy, resulting in inadequately protected flocks. Live THEV vaccines may exhibit immunosuppressive properties, predisposing to secondary bacterial infections [67]. 

Additional factors that could reduce the efficacy of vaccination include the presence of immunosuppressive infectious agents hampering the development of vaccine-induced immunity [68]. Experimental infection of turkeys with avian metapneumovirus (aMPV) also results in the diminished efficacy of THEV vaccines [69]. 

Despite the testing of experimental subunit and viral-vectored vaccines, these approaches have not been adopted in practical field applications. Viral DNA can persist in various tissues for at least 15 weeks post-virus inoculation. Nonetheless, flock seropositivity after vaccination may decrease to 83% toward the conclusion of the fattening period. Under such circumstances, inadequately protected flocks may remain susceptible to THEV infections [69].

Regarding vaccination against THEV, avirulent strains of THEV or Marble spleen disease virus (MSDV) have been used successfully as viable vaccines for some time [70,71]. The live attenuated vaccines currently available are prepared from spleen homogenates obtained from 6-week-old SPF turkeys inoculated with the virulent strain of THE or a strain produced in vitro using RP19 cells [70]. Nevertheless, the latter are currently unavailable in Europe. It is crucial to emphasize that vaccines designed for administration to turkeys should be avoided in pheasants and, conversely, vaccines meant for pheasants should not be used in turkeys. This is because avirulent isolates employed for immunizing one species tend to exhibit virulence when introduced into the other. In Europe, there are currently two types of vaccines available for controlling Turkey Hemorrhagic Enteritis:

I. Live-attenuated autogenous vaccine: This vaccine is produced by infecting 6-week-old female Specific Pathogen-Free (SPF) turkeys with the Domermuth strain (Virginia Avirulent Strain) [39]. The administration is carried out via drinking water, and the vaccination starts when the turkeys reach 4 weeks of age. Currently, these vaccines are widely used in the United States and some European countries. Vaccinated flocks demonstrating 60% or higher seroconversion with splenic homogenate indicate full protection. For flocks with low seroconversion, particularly when cell culture-based vaccines are used, a second vaccination should be administered one week after the first. The presence of immunosuppressive agents such as aMPV or residual water sanitizers in the pipeline can reduce vaccination efficacy [69].
II. Inactivated vaccine: The production of this vaccine involves the infection of female turkeys in the shed and then the removal of their spleens to quantify and inactivate the virus. The inactivated vaccine is mixed with liquid paraffin and administered through a subcutaneous injection in the middle third of the neck region. The initial immunization is administered at 3–4 weeks of age, with the subsequent vaccination administered at 7–8 weeks of age.

A third approach to vaccine production involves the cultivation of non-virulent TAdV-3 in peripheral blood leukocytes, a method employed in the USA and Canada. In Canada, the tissue culture vaccine is the only one approved for THEV control (Table 2). This vaccine is administered in a single full dose (≥102.6 TCID50) between 3.5 and 6 weeks of age or in two doses with a lower quantity (e.g., 2/3 of a dose or ≥102.6 TCID50) at days 25 and 35. This strategy is intended to reduce the circulation of THEV in the field among susceptible birds to a very low level. The first vaccination on day 25 is targeted at birds with low maternal antibodies, while the second vaccination on day 35 is targeted at those not immunized during the first cycle, probably due to high levels of neutralizing maternal antibodies or poor vaccine uptake [4]. The challenge of distinguishing between THEV vaccine and field strains is recognized due to the high level of nucleotide sequence similarity, reaching 99.9%, between virulent and avirulent THEV strains [35,72,73].

Quaglia et al. [74] showed that the analysis of the 3′ region sequence of the ORF1 gene is a valuable method for characterizing THEV strains at the molecular level and distinguishing between vaccine-like and field strains. In this study, a total of 80 samples, previously confirmed as positive for THEV, were included and underwent analysis through sequencing and phylogenetic techniques. This analysis utilized a new set of PCR primers that targeted a specific genomic area encompassing the partial ORF1, complete hyd, and partial IVa2 gene sequences. Furthermore, the analysis involved the Dindoral SPF commercial live vaccine. The eight strains of THEV originating from Italy exhibited three distinct mutations within the 3’ section of the ORF1 gene. These mutations were not found in the Dindoral SPF vaccine strain, suggesting that this diagnostic tool should be regularly employed to enhance control strategies and ensure accurate diagnoses [74]. 

The effective prevention and control of HE begins by following the best management practices, particularly focusing on the implementation of biosafety protocols. In multi-age herds, achieving complete elimination of the virus is considered impractical. In these situations, vaccination is the only effective method of controlling and preventing the disease.

## 11. Conclusions and Future Perspectives

THEV infection is characterized by depression, gastrointestinal hemorrhage, and transient immunosuppression, followed by increased mortality due to blood loss and secondary infections, resulting in substantial economic losses. Over the years, the incidence of clinical manifestations has decreased due to vaccination and the circulation of avirulent strains in the field. However, it should be noted that avirulent strains are also capable of triggering subclinical disease by causing immunodepression, leading to secondary bacterial infections. Previous research suggests that the genetic variability of THEV in the field may not be as low as previously thought. Some sequences indicate that adaptive changes, potentially driven by increased vaccine pressure, have occurred and may facilitate immune evasion (e.g., BC strains—fib knob domain gene). However, since vaccination plans have been implemented, outbreaks on turkey farms have become rare, contributing to a reduction in antibiotic use due to secondary infections. Clinical outbreaks are probably associated with a poor vaccination response. The economic importance derives mainly from secondary infections. Therefore, the rigorous implementation of biosecurity standards and practices is crucial to control the spread of the virus and minimize mortality.

## Figures and Tables

**Table 1:** Virus classification and affected species [14,15].

Genus	Species	Species of Host Affected	Disease
*Aviadenovirus* (Group I Adenoviruses)	Fowl adenovirus (FAdV) Goose adenovirus (GoSdV) Duck adenovirus B (DAdV 2) Pigeon adenovirus B (PiAdV 2) Turkey adenovirus B (TAdV)	Chickens, turkeys, ducks, geese, pigeons, wild species.	Inclusion body hepatitis, hydropericardium syndrome, gizzard erosions. Adenovirus infection in duck, pigeon, and turkey.
*Siadenovirus* (Group II Adenoviruses)	Turkey adenovirus A (TAdV 3)	Chickens, turkeys, pheasants	Hemorrhagic enteritis (turkey), Marble spleen disease (Pheasant), Avian adenovirus splenomegaly (chicken).
*Atadenovirus* (Group III Adenoviruses)	Duck adenovirus A (DAdV-1)	Ducks, bovine, ovine, deer, possums, snakes	Egg drop syndrome

**Table 2:** Live vaccines available in Europe, the USA, and Canada for THEV control and prevention (the results presented in this table are derived from a search conducted on Animalytix.com).

Commercial Name	Company	Country	Strain	Administration Route	Age	Species
Dindoral	Boehringer Ingelheim (Germany)	Europe	Domermuth (Marble Spleen Disease avirulent virus)	Drinking water	From the 4th week	Turkeys, Pheasants
Hemorrhagic enteritis vaccine,	Hygieia (Canada)	USA	Type 2 avian adenovirus of pheasant origin	Drinking water	From the 5th week	Turkeys
H.E. Vac	Arko (USA)	USA; Canada	Type II avian adenovirus is propagated in a lymphoblastoid cell line (MDTC-RP19)	Drinking water	At thirty days	Turkeys
Oralvax-HE	MSD AH (USA& Canada)	USA; Canada	Avirulent Type II avian adenovirus of pheasant origin	Drinking water	6 weeks or older	Turkeys
Pro’tect Hemorrhagic enteritis vaccine	Brinton (USA)	USA	Live cell culture-grown virus	Drinking water	22 days or older	Turkeys
Adenomune ll	Ceva (Canada)	USA	Live avirulent strain of hemorrhagic enteritis virus of pheasant origin	Drinking water	5 weeks or older	Turkeys

## Data Availability

Not applicable.
